# Genetic Analysis of Intraductal Carcinoma of the Prostate Detected in High-Grade Prostatic Intraepithelial Neoplasia Cases

**DOI:** 10.7759/cureus.76165

**Published:** 2024-12-21

**Authors:** Ryuta Watanabe, Noriyoshi Miura, Mie Kurata, Riko Kitazawa, Tadahiko Kikugawa, Takashi Saika

**Affiliations:** 1 Department of Urology, Ehime University Graduate School of Medicine, Toon, JPN; 2 Department of Analytical Pathology, Ehime University Graduate School of Medicine, Toon, JPN; 3 Department of Diagnostic Pathology, Ehime University Hospital, Toon, JPN

**Keywords:** basal cell staining, genetic analysis, high-grade prostatic intraepithelial neoplasia, hr gene mutations, intraductal carcinoma of the prostate

## Abstract

Background

The accurate diagnosis of intraductal carcinoma of the prostate (IDC-P) is occasionally challenging due to the similarity in pathological morphology between IDC-P and high-grade prostatic intraepithelial neoplasia (HGPIN). In this report, we reviewed the pathology of cases previously diagnosed as HGPIN to search for IDC-P cases effectively. In addition, we examined whether those cases had genetic abnormalities.

Methods

We reviewed 98 patients with HGPIN who underwent prostatectomy at our hospital between 2011 and 2021. They were reviewed by three pathologists to search for IDC-P findings by adding immunostaining for basement membrane markers. Genetic testing of prostatectomy specimens was performed to identify the presence of gene mutations.

Results

The typical IDC-P was diagnosed in two of the 98 patients. The Gleason score of background prostate cancer (PCa) was 4+5 and 4+4. Genetic testing revealed several mutations in DNA repair-related genes, such as *CHEK2, FANCC, TOE1, RECQL, USG2A*, and *PRPF31*. The pathological significance of these mutations has conflicting interpretations, as referenced in the ClinVar.

Conclusions

IDC-P cases can be identified from past HGPIN cases, and cases with genetic abnormalities of conflicting pathological significance can be efficiently detected. Accurate diagnosis of IDC-P enables early intervention with precision medicine for PCa. It is useful to pay attention to HGPIN cases to avoid missing true IDC-P.

## Introduction

Intraductal carcinoma of the prostate (IDC-P) is a pathological entity in which atypical cells from outside the glandular ducts invade and proliferate in the normal glandular architecture, with partial preservation of basal cells, showing cribriform and extensive growth patterns. It has recently attracted attention as a finding associated with poor prognosis. The presence of IDC-P observed in radical prostatectomy is associated with a higher Gleason score, larger tumor volumes, extra-prostatic extension, squamous cell carcinoma at the resection margin, and accelerated disease progression [1-5].

According to reports from Western countries, in addition to the* TMPRSS2-ERG* fusion gene, *TP53, RB1,* and *PTEN* deletions; HR (homologous recombination) gene mutations, including *BRCA* mutations; are frequently detected in patients with IDC-P [6-9]. Interestingly, one study has suggested that *BRCA2* mutations are detected in approximately 42% of IDC-P cases [10].　Therefore, promoting precision medicine through accurate diagnosis of IDC-P is crucial.

IDC-P represents the invasion of adenocarcinoma into normal glandular structures and is diagnosed based on the presence of highly dysplastic tumor cells in cribriform or solid structures. In contrast, high-grade prostatic intraepithelial neoplasia (HGPIN) is defined by the presence of cells with a neoplastic appearance that resides within existing acini or ducts and is enclosed by the surrounding basal cell layer, and may be a precursor to invasive carcinoma. IDC-P and HGPIN share similarities in preserving the basal cell layer and exhibiting atypical cell proliferation in the acini, making it challenging to distinguish between them. Therefore, in this study, we retrospectively investigated the presence of IDC-P in patients initially diagnosed with HGPIN surrounding high-grade adenocarcinoma. Furthermore, we examined the presence of gene mutations using genetic testing.

## Materials and methods

We conducted a retrospective study of patients who underwent total prostatectomy (radical retropubic prostatectomy (RRP), laparoscopic radical prostatectomy (LRP), robot-assisted laparoscopic prostatectomy (RALP)) for prostate cancer (PCa) at our hospital between November 2011 and November 2021. From the eligible 1026 patients, we selected 98 patients with PCa that included HGPIN. We paid particular attention to cases where there was a proliferative tumor in the normal glandular duct.

IDC-P is defined as a pathological entity in which atypical invasive cancer cells outside the ducts invade and proliferate in normal ductal structures, and is characterized by the partial persistence of basal cells and a cribriform and extensive growth pattern. Therefore, immunohistological tests were performed to detect basal cells.

Immunohistochemistry was carried out on surgical tissue samples that had been fixed in 10% neutral buffered formalin and embedded in paraffin. Sections were prepared using a microtome at a thickness of 3-5 μm and stained following standard procedures. For the detection of basal cells, a *p63* antibody combined with a high molecular weight (HMW) cytokeratin antibody, sourced from Proteintech (Rosemont, USA), was utilized. Nuclear staining was performed using Hoechst 33342 (1:2000 dilution; Molecular Probes). Only when the basal cells were unclear and it was necessary to distinguish them from invasive carcinoma, we performed basal cell staining. The pathology reports were reviewed by three pathologists from Ehime University Hospital, including a U.S.-trained pathologist with expertise in IDC-P diagnosis, to identify cases indicative of IDC-P and assess for relevant diagnostic markers.

DNA was extracted from resected prostate formalin-fixed paraffin-embedded (FFPE) samples using the QIAamp DNA FFPE Tissue Kit (Qiagen, Hilden, Germany). Then, the DNA was analyzed using next-generation sequencing (Axen *BRCA* Premium; Macrogen, Korea) for the presence of HR gene mutations, including *BRCA* mutations, in both cases. The list of genes that can be detected with this kit is as follows; *Rad51D, Rad51C, RP1, Rad51B, NBN, FAM175A, MLH1, APOBEC3G, PTEN, APOBEC3B, EPCAM, MEN1, FANCM, APOBEC3A, MUTYH, PMS2, USH2A, FANCC, PRPF31, RET, RPGR, POLE, RINT1, TP53, RB1, MFN2, SLC26A4, RECQL, PMP22, BLM, STK11, CDH1, CHEK2, POU3F4, PALB2, APC, TECTA, MSH6, MPZ, MSH2, VHL, NF1, BRCA2, BRIP1, ATR, PRPH2, ATM, GJB1, BRCA1, GJB2, POLD1, RHO, BARD1*

This study was approved by the Institutional Review Board of Ehime University Hospital (Approval No. 2205001).

## Results

**Figure 1:**
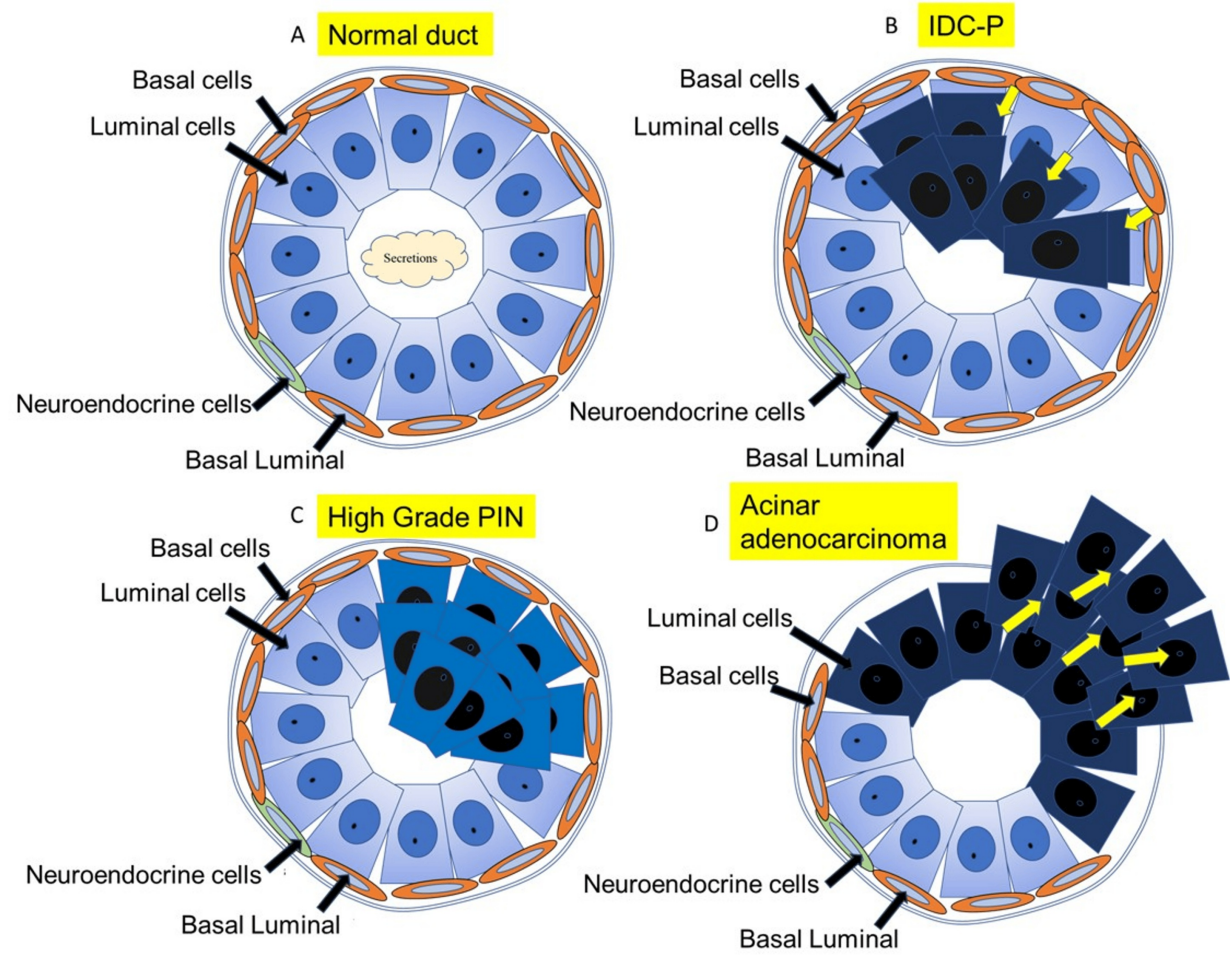
Pathological schema of various stages of prostate cancer. The normal glandular duct structure of the prostate consists of luminal, basal, neuroendocrine, and basal luminal cells (A). Intraductal carcinoma of the prostate (IDC-P) is a pathological pattern in which atypical cells from outside the glandular ducts invade, proliferate, and conduit in the normal gland where basal cells are partially preserved, showing cribriform and extensive growth (B). High-grade prostatic intraepithelial neoplasia (HGPIN), characterized by atypical cell proliferation within a pre-existing adenoma or adenocarcinoma, occurs within the glandular ducts (C), whereas adenocarcinoma is invasive outside the glandular ducts (D). Figure credit: Ryuta Watanabe.

IDC-P is a pathological condition in which tumor cells outside of normal glandular ducts invade and proliferate in normal glandular tissue. IDC-P is differentiated from HGPIN and acinar carcinoma, which occur within normal glandular ducts. IDC-P is characterized by preserved basal cells despite the high degree of tumor atypia (Figure 1). Because both HGPIN and IDC-P retain basal cells, it requires experience as a pathologist to differentiate between the two.

**Figure 2:**
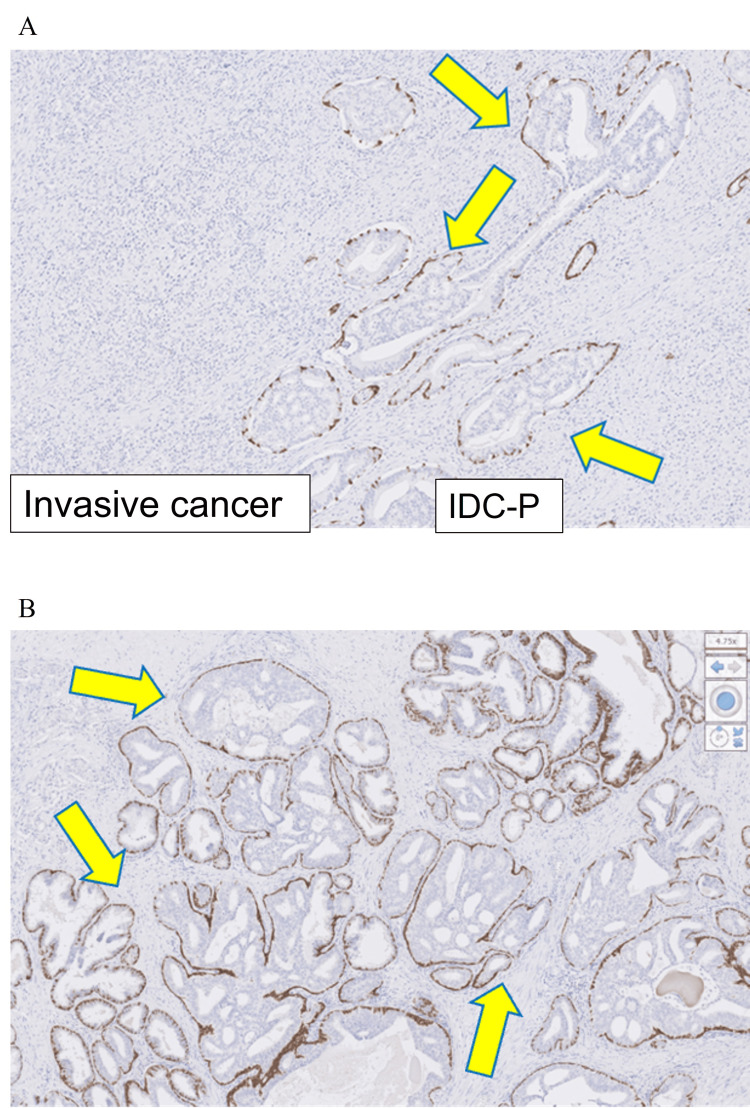
Pathology report findings at initial diagnosis with high-grade prostatic intraepithelial neoplasia (HGPIN) and immunohistochemical staining images of two cases diagnosed with intraductal carcinoma of the prostate (IDC-P) (A) Case 1: Gleason score 4+5 contains a prostatic intraepithelial neoplasia component around the tumor (Yellow arrows). (B) Case 2: Atypical cells with distinct nucleoli on a background of prostatic intraepithelial neoplasia infiltrate and proliferate in the form of small and fused glandular ducts with an indistinct lumen (Yellow arrows). Figure credit: Ryuta Watanabe.

IDC-P was diagnosed in two of the 98 patients (Figure 2). All of the pathology reports indicated the presence of HGPIN, suggesting tumor growth within the ducts. IDC-P and HGPIN share cytological features such as nuclear enlargement, increased chromatin, and enlarged nucleoli. According to Guo and Epstein, the main characteristics of IDC-P are as follows: 1) frequent solid or high-density cribriform patterns, 2) marked nuclear enlargement (six times that of normal cells), 3) may show marked atypia, 4) frequent mitotic figures in ducts/acini, and 5) frequent focal necrosis, and they incorporated these criteria into their diagnosis [11]. In these two cases, the diagnosis of IDC-P was made because of the marked nuclear enlargement, which is characteristic of HGPIN, and the expansion of the glandular ducts due to tumor growth within the normal ducts, as well as the presence of structures resembling the cribriform pattern. The presence of residual basal cell layers was confirmed by immunohistochemical staining, and this was useful for differentiating IDC-P from prostate invasive carcinoma.

The Gleason scores of the PCa in the background were 4+5 and 4+4. It was thought that the surrounding invasive cancer had retrogradely flowed into the normal ducts. At present, 28 and 37 months have passed, respectively, and both patients have progressed without recurrence.

Genetic testing revealed multiple mutations in *CHEK2, FANCC, TOE1, RECQL, USH2A*, and *PRPF31. CHEK2* is involved in DNA repair in response to DNA damage, cell cycle arrest, and apoptosis [12-13]. The *FANCC* gene is involved in inter-strand cross-link repair and the maintenance of normal chromosome stability [14-16]. *TOE1* is involved in RNA phosphodiester bond hydrolysis, exonucleolytic, and snRNA 3'-end processing [17]. *RECQL* is a member of the DNA helicase family, and DNA helicases are enzymes that are involved in various DNA repair processes, including mismatch repair, nucleotide excision repair, and direct repair [18]. *USH2A* codes for multiple usherin isoforms due to an alternative splicing [19-20]. *PRPF31 *is a gene that encodes the splicing factor hPRP31 [21].

However, when referring to ClinVar for the pathological significance of these variants, the interpretation is currently divided. (Table 1).

**Table 1:** Clinical characteristics and genetic analysis of two patients with intraductal carcinoma of the prostate (IDC-P) The Gleason score of background prostate cancer (PCa) was 4+5 and 4+4. Both patients had progressed without recurrence. Genetic testing revealed several mutations in *CHEK2, FANCC, TOE1, RECQL, USG2A*, and *PRPF31*, although their pathological significance has conflicting interpretations as referenced in the ClinVar. iPSA: initial PSA (prostate specific antigen) level

	Age	iPSA (ng/ml)	Gleason Score	TNM classification	Follow up periods	Recurrence	Gene abnormality			
Case 1	61	31.5	4+5	pT2bN0M0	28 months	None	TOE1 (5 prime UTR) c.-45G>A -	FANCC (synonymous) c.1494T>C p.Ala498Ala	CHEK2 （missense） c.538C>T p.Arg180Cys	ー
Case 2	73	5.5	4+4	pT2aN0M0	37 months	None	RECQL （missense） c.1382A>G p.His461Arg	TOE1 (5 prime UTR) c.-45G>A -	USH2A (synonymous) c.879T>G p.Leu293Leu	PRPF31 (synonymous) c.510C>T p.Thr170=

## Discussion

Epstein's criteria are commonly used to diagnose IDC-P, which is morphologically divided into two main categories: those with a cribriform pattern or comedo necrosis and those that resemble HGPIN but show high cellular atypia. In this study, we searched for IDC-P among cases previously diagnosed with HGPIN, mainly focusing on the latter pathological pattern. It should be noted that the basement membrane structure and basal cell staining are essential for diagnosing both IDC-P and HGPIN [22].

**Figure 3:**
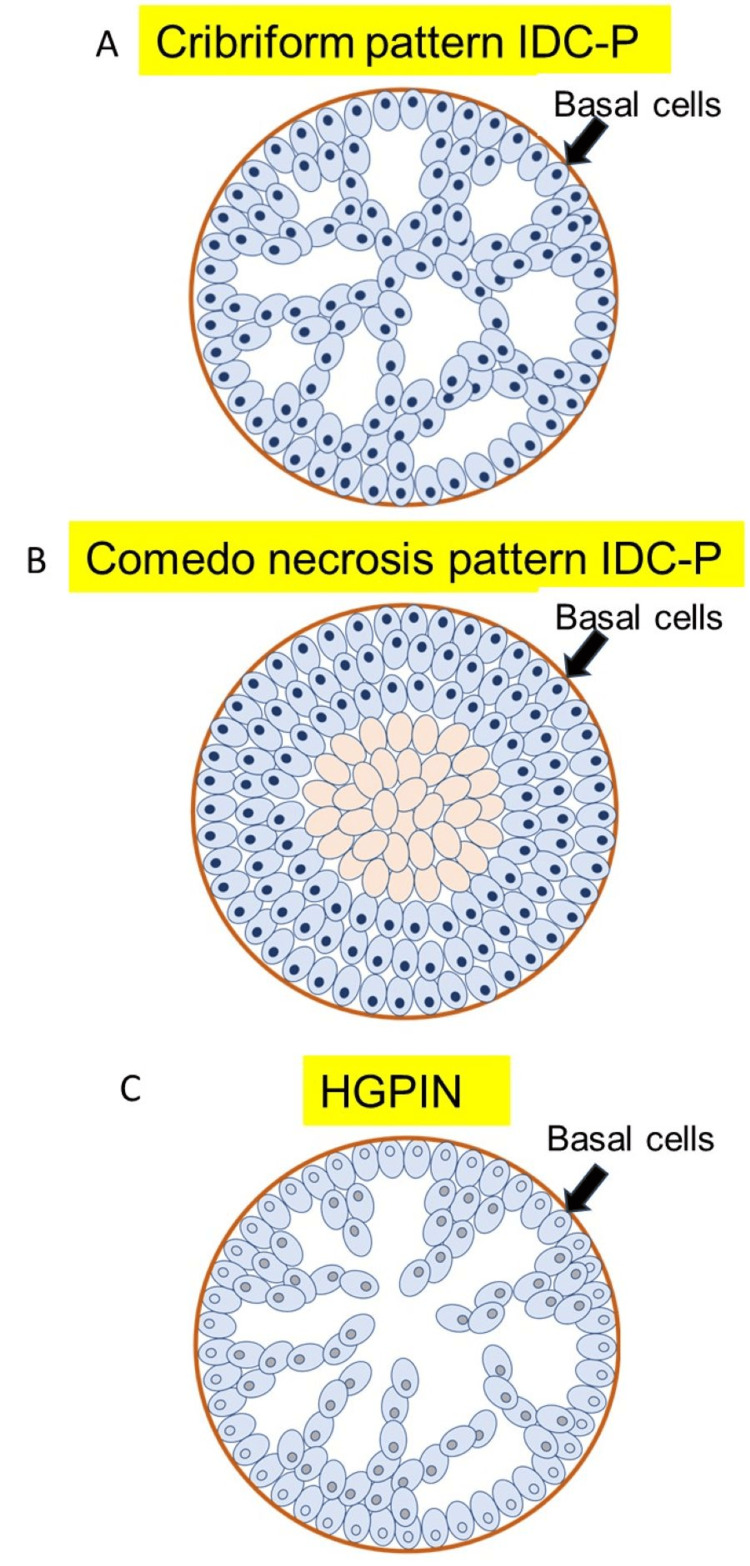
Differentiation between intraductal carcinoma of the prostate (IDC-P) and high-grade prostatic intraepithelial neoplasia (HGPIN) based on structural patterns in normal glandular ducts surrounded by basal cells IDC-P often shows strong nuclear atypia of cells and a cribriform/comedo necrosis pattern (A and B), whereas HGPIN often shows weak nuclear atypia of cells and a finger-like cell proliferation pattern (c). Basal cells are preserved in both cases.

HGPIN is a precancerous lesion characterized by a proliferation of atypical cells within the prostate glands and acini, with enlarged nuclei and distinct nucleoli. In contrast, IDC-P is characterized by a cribriform growth pattern, markedly enlarged nuclei (six times the size of normal cells), marked pleomorphism, often mitotic activity, and comedo necrosis (Figure 3) [11].

There is still debate about whether IDC-P with high-grade adenocarcinoma should be included in the Gleason score [23]. The clinical significance of IDC-P in these two cases involving high-grade adenocarcinoma is still unclear. However, our approach, which focuses on HGPIN, can be an effective option for obtaining IDC-P cases. It should be noted that obvious IDC-Ps with these remarkable characteristics are rarely encountered, and it is often difficult to diagnose IDC-Ps in borderline pathology. In some cases, immunochemical staining of basal cells may be useful for accurate IDC-P diagnosis.

IDC-P arises from the same ancestral clone as adenocarcinoma, but specific abnormalities, such as *MYC* amplification, occur earlier in *BRCA2*-mutant PCa. Therefore, searching for IDC-P cases may lead to early diagnosis of genetic abnormalities and early gene therapy in the future. It will also play an important role in recognizing potential carriers of germline variants associated with cancer predisposition syndromes. Although many variants were detected in the two cases, their pathological significance has conflicting interpretations [24]. One possibility is that the patients were in good condition and did not have an increased number of important somatic gene abnormalities. Identifying important genetic abnormalities in cases with progressive outcomes or by performing single-cell analysis may be possible.

The limitations of this study include the following: (1) We have not reviewed cases that do not include HGPIN; (2) Even if HGPIN is present, not all cases were included in the pathology report; (3) Some IDC-Ps do not present with HGPIN-like findings; (4) We did not compare cases with and without HGPIN diagnosis; and (5) IDC-P associated with low Gleason grade adenocarcinoma was not included in this study.

Nevertheless, our study demonstrated that focusing on HGPIN cases can effectively identify IDC-P cases, which is particularly useful given the challenges of diagnosing IDC-P and its rarity in many institutions. It is necessary to be aware that the initial finding of HGPIN includes IDC-P cases with a possible poor prognosis and high clinical importance. Accurate diagnosis of IDC-P cases, considering the differentiation between HGPIN and IDC-P, will be useful in treating patients. We hope this report will raise awareness about IDC-P diagnosis and more patients will benefit from precision medicine through early genetic diagnosis.

## Conclusions

We identified two cases of IDC-P from a retrospective search of 98 cases of adenocarcinoma with HGPIN. As HGPIN and IDC-P are similar at first glance, there is a possibility that there are cases of IDC-P that have been misdiagnosed as HGPIN in the past. In both cases, multiple HR gene mutations of unknown pathological significance were detected. Accurate diagnosis of IDC-P cases is expected to enable early intervention through personalized medicine for prostate cancer by efficiently identifying cases with genetic abnormalities. Based on our findings, the challenge for the future is to achieve accurate diagnosis by raising awareness of the characteristic pathological findings of IDC-P.
